# Diagnostic performance of artificial intelligence to detect genetic diseases with facial phenotypes: A protocol for systematic review and meta analysis

**DOI:** 10.1097/MD.0000000000020989

**Published:** 2020-07-02

**Authors:** Bosheng Qin, Qiyao Quan, Jingchao Wu, Letian Liang, Dongxiao Li

**Affiliations:** a College of Information Science and Electronic Engineering; b College of Computer Science and Technology; c College of Electrical Engineering, Zhejiang University, Hangzhou, China.

**Keywords:** artificial intelligence, deep learning, diagnostic performance, facial phenotypes, genetic diseases, machine learning

## Abstract

**Background:**

Many genetic diseases are known to have distinctive facial phenotypes, which are highly informative to provide an opportunity for automated detection. However, the diagnostic performance of artificial intelligence to identify genetic diseases with facial phenotypes requires further investigation. The objectives of this systematic review and meta-analysis are to evaluate the diagnostic accuracy of artificial intelligence to identify the genetic diseases with face phenotypes and then find the best algorithm.

**Methods:**

The systematic review will be conducted in accordance with the “Preferred Reporting Items for Systematic Reviews and Meta-Analyses Protocols” guidelines. The following electronic databases will be searched: PubMed, Web of Science, IEEE, Ovid, Cochrane Library, EMBASE and China National Knowledge Infrastructure. Two reviewers will screen and select the titles and abstracts of the studies retrieved independently during the database searches and perform full-text reviews and extract available data. The main outcome measures include diagnostic accuracy, as defined by accuracy, recall, specificity, and precision. The descriptive forest plot and summary receiver operating characteristic curves will be used to represent the performance of diagnostic tests. Subgroup analysis will be performed for different algorithms aided diagnosis tests. The quality of study characteristics and methodology will be assessed using the Quality Assessment of Diagnostic Accuracy Studies 2 tool. Data will be synthesized by RevMan 5.3 and Meta-disc 1.4 software.

**Results:**

The findings of this systematic review and meta-analysis will be disseminated in a relevant peer-reviewed journal and academic presentations.

**Conclusion:**

To our knowledge, there have not been any systematic review or meta-analysis relating to diagnosis performance of artificial intelligence in identifying the genetic diseases with face phenotypes. The findings would provide evidence to formulate a comprehensive understanding of applications using artificial intelligence in identifying the genetic diseases with face phenotypes and add considerable value in the future of precision medicine.

**OSF Registration:**

DOI 10.17605/OSF.IO/P9KUH.

## 1 Introduction

Genetic Diseases affect a majority of the population during their lifetime.^[1]^ It was reported that this kind of diseases affects nearly 8% of the population.^[2]^ Many affected patients present signs and symptoms will affect their lifelong health status and quality of life.^[3,4]^ Early diagnosis is necessary to generalize to prevent the occurrence of potential health problems, such as critical respiratory problems, cardiovascular dysfunction, developmental delays, and mental retardation.^[5]^ It can also benefit the patients for lifelong health care involving cardiac, physical, speech, and neurological therapies.^[3]^

Many genetic syndromes are known to have distinctive facial phenotypes, which are highly informative to provide an opportunity for automated detection.^[6–9]^ Recent advances in artificial intelligence involving computer vision present the opportunity for development in many fields. The performance of tasks such as object localization, detection, recognition, and segmentation based on public datasets has dramatically improved.^[10]^ In medicine, artificial intelligence has demonstrated significant advantages in disease diagnosis and lesion segmentation due to its great capacity for feature extractions.^[11,12]^ The distinctive facial characteristics of genetic diseases with facial phenotypes might provide an opportunity for automatic identification.^[13–20]^ In recent years, artificial intelligence has been developed for the automated and accurate identification of various genetic diseases with facial phenotypes using 2-dimensional or 3-dimensional facial images.^[5,9,21–25]^

However, the diagnostic performance of different algorithms base on artificial intelligence to identify genetic diseases with facial phenotypes requires further investigation. A meta-analysis of diagnostic performance represents a powerful method to summarize findings in the publications by considering and enabling synthesis of differences between various studies. Therefore, the objectives of this review and meta-analysis are to evaluate the diagnostic accuracy of artificial intelligence in identifying the genetic disease with face phenotypes and then find the best algorithm.

## 2 Methods

The systematic review and meta-analysis will be performed in accordance with the “Preferred Reporting Items for Systematic Reviews and Meta-Analyses Protocols” guidelines. The protocol has been registered in the Open Science Framework (OSF) with an identification number of DOI 10.17605/OSF.IO/P9KUH. The possible update after publication will also be disclosed in the OSF registration. Formal ethical approval is not required since this systematic review is a synthesis and analysis of secondary data based on previous published studies.

### 2.1 Search strategy

Searched electronic databases will involve: PubMed, Web of Science, IEEE, Cochrane Library, Ovid, EMBASE, and China National Knowledge Infrastructure for reports on the diagnostic performance of artificial intelligence on genetic diseases published between 1989 and April 2020. For the specialty-specific meta-analysis, the fields of genetics, pediatrics, and computer science will be chosen, as they have some studies with available data. Two reviewers will independently screen and select the titles and abstracts of the studies retrieved during the database searches and perform full-text reviews and extract related data. Disagreements regarding inclusion of studies will be resolved by discussion with a third reviewer.

The comprehensive computer-based literature search will be conducted to identify all relevant diagnostic performance for genetic diseases with facial phenotypes based on artificial intelligence. The keywords or MeSH terms of the searching strategies are “artificial intelligence”, “computer-aided”, “deep learning”, “machine learning“, ”neural networks“, ”facial images“, ”facial recognition“, ”automatic diagnosis“, ”image processing“, or ”genetic disease“. All the publications will be searched by 2 reviewers independently.

### 2.2 Inclusion criteria and exclusion criteria

#### 2.2.1 Inclusion criteria

Studies in English or Chinese that comprise a diagnostic accuracy assessment of artificial intelligence algorithms, as used facial images in human populations, will be eligible for inclusion. Only studies that provide either diagnostic accuracy raw data or accuracy, recall, specificity, precision will include in the meta-analysis.

#### 2.2.2 Exclusion criteria

Studies were not written in English or Chinese. Letters, abstracts, case studies, reviews, and animal studies will not be considered.

### 2.3 Study selection and data extraction

The protocol of selection process is summarized with the flow diagram in accordance with the Preferred Reporting Items for Systematic Reviews and Meta-Analyses Protocols framework. References from the above search strategy will be transferred to Endnote 9.2 for English articles and NoteExpress 3.2 for Chinese articles. Two reviewers will independently extract demographic and diagnostic accuracy data from the selected studies using a predefined electronic data extraction spreadsheet. Data will be listed as

(1) first author;
(2) publication years;
(3) name of genetic diseases
(4) algorithm;
(5) datasets;
(6) diagnostic accuracy raw data - positives and negatives, true and false;
(7) accuracy, specificity, precision, recall, F1 score, Matthias correlation coefficient (MCC) scores, or quadratic weight κ. If the available data permits, the binary diagnostic accuracy data will be extracted and constructing contingency charts consisting of true-negative, false-negative, true-positive, and false-positive.

### 2.4 Quality evaluation

The biases of involved studies will be assessed by 2 reviewers using the Quality Assessment of Diagnostic Accuracies Studies 2 checklist which consists of 4 dimensions: patient selection, reference standard, index test, and timing and flow. The risk of bias in each dimension could be classified as “High”, “Low”, or “Unclear” risk from different points. Any disagreement that arises between 2 reviewers will be resolved by discussion. Studies with high risks of biases will be considered for exclusion.

### 2.5 Data analysis and synthesis

The primary outcome measures include diagnostic accuracy, as defined by accuracy, recall, specificity, and precision. If necessary, area under the precision-recall curve and area under the receiver operating characteristic curves will also be measured with the report of the precision-recall curve and receiver operating characteristic curve. The summary receiver operating characteristic curves and descriptive forest plot will be used to represent the performance of a diagnostic examination. We will plot the prediction regions and 95% CI around the averaged accuracy estimates in the summary receiver operating characteristic space, and the area under the receiver operating characteristic curve will be calculated. Heterogeneity among included studies will be checked using I^2^ test. Subgroup analysis will be performed for different algorithms aided diagnosis tests. Data will be synthesized by the software of RevMan 5.3 and Meta-disc 1.4.

## 3 Discussion

Recent advances in artificial intelligence with computer vision, involving machine learning and specifically deep learning, present the opportunity for novel systems in many fields. The facial phenotypes of genetic diseases are critical for syndrome diagnosis, and thus facial analysis using computer vision has great potential. Although artificial intelligence has been developed for the automatic and accurate identification of genetic diseases with facial phenotypes using facial images, the diagnostic performance of different algorithms based on artificial intelligence have not been investigated. To the best of our knowledge, there have not been any review articles in the diagnosis performance using artificial intelligence to detect genetic disease with facial phenotypes. Therefore, we intend to conduct evaluation systematically for the related studies, and the findings would add considerable value in the future of precision medicine.

There are several limitations to this study. This systematic review would be highly dependent on the quality of the original studies in case of retrospective researches. Convenience sampling and small sample size in original studies have contributed to some sampling bias, which could limit the generalization of the results.

## Author contributions

**Analysis planning:** Bosheng Qin, Qiyao Quan

**Conceptualization:** Bosheng Qin, Dongxiao Li

**Data curation:** Bosheng Qin, Qiyao Quan, Jingchao Wu

**Draft manuscript:** Bosheng Qin

**Investigation:** Bosheng Qin, Letian Liang, Qiyao Quan

**Manuscript editing:** Bosheng Qin, Dongxiao Li

**Methodology:** Bosheng Qin, Jingchao Wu
